# *Cavisoma magnum* (Cavisomidae), a unique Pacific acanthocephalan redescribed from an unusual host, *Mugil cephalus* (Mugilidae), in the Arabian Gulf, with notes on histopathology and metal analysis

**DOI:** 10.1051/parasite/2018006

**Published:** 2018-02-09

**Authors:** Omar M. Amin, Richard A. Heckmann, Majid A. Bannai

**Affiliations:** 1 Institute of Parasitic of Diseases, 11445 E. Via Linda # 2-419, Scottsdale, AZ. 85259 USA; 2 Department of Biology, Brigham Young University, 1114 MLBM, Provo, UT 84602 USA; 3 Marine Vertebrate, Marine Science Center, University of Basrah, Basrah Iraq

**Keywords:** Acanthocephala, *Cavisoma magnum*, *Mugil cephalus*, *Chanos chanos*, Arabian Gulf, redescription, SEM, histopathology, metal analysis

## Abstract

*Cavisoma magnum* (Southwell, 1927) Van Cleave, 1931 was originally described from a sea bass, *Serranus* sp. and spotted surgeonfish, *Ctenochaetus strigosus* (Perciformes) off Sri Lanka before its more recent redescription from milkfish in the Philippines in 1995. These reports were based on only light infections of their host fishes. Of the few flathead grey mullets, *Mugil cephalus* (Mugilidae), that we examined in the Arabian Gulf, one fish was infected with 1,450 worms. One milkfish, *Chanos chanos* (Chanidae), from the same location in the Arabian Gulf, was also heavily infected with specimens of *C. magnum.* The descriptions of this unique large worm are revised and for the first time, we provide SEM images, new systematic observations, metal analysis of hooks showing extremely high levels of sulfur, and histopathology in the mullet intestinal tissue. Adjustments and corrections of previous descriptive accounts are made. The histopathology studies show extensive damage to the host intestinal tissue including epithelial necrosis, hemorrhaging and worm encapsulation. There is an extensive amount of host connective tissue surrounding the worm. Results of x-ray analysis displayed high levels of sulfur in proboscis hooks, especially at the tips and edges of these attachment structures.

## Abstract (fr)

*Cavisoma magnum* (Southwell, 1927) Van Cleave, 1931 a été décrit à l’origine à partir de spécimens parasites d’un *Serranus* sp. et du poisson-chirurgien *Ctenochaetus strigosus* (Perciformes) au Sri Lanka avant sa redescription plus récente à partir de spécimens parasites du chano aux Philippines, en 1995. Ces rapports étaient basés uniquement sur des infections légères de leurs poissons-hôtes. Parmi les quelques mulets à grosse tête, *Mugil cephalus* (Mugilidae) que nous avons examinés dans le golfe Persique, un poisson était infecté par 1 450 vers. Un chano, *Chanos chanos* (Chanidae), originaire du même endroit dans le golfe Persique, était également fortement infecté par des spécimens de *C. magnum*. Les descriptions de ce grand ver sont révisées et pour la première fois, nous fournissons des images en MEB, de nouvelles observations systématiques, des analyses des crochets montrant des niveaux extrêmement élevés de soufre, et l’histopathologie dans le tissu intestinal du mulet. Des ajustements et corrections des descriptions précédentes sont effectués. Les études histopathologiques montrent des dommages importants du tissu intestinal de l’hôte, y compris nécrose épithéliale, hémorragie et encapsulation des vers. Une grande quantité de tissu conjonctif de l’hôte entoure le ver. Les résultats de l’analyse par rayons X ont montré des niveaux élevés de soufre dans les crochets du proboscis, en particulier aux extrémités et aux bords de ces structures de fixation.

## Introduction

*Cavisoma magnum* (Southwell, 1927) Van Cleave, 1931 was originally described as *Oligoterorhynchus magnus* by Southwell [18] from the stomach and pyloric ceca of the sea bass, *Serranus* sp. Cuvier (Serranidae) (20 worms) and from another bass, the spotted surgeonfish *Acanthurus strigosus* Bennett (=*Ctenochaetus strigosus* Bennet) (Acanthuridae) (6 worms) off Negapatam, Ceylon (Sri Lanka). Van Cleave [20] assigned it to the new genus *Cavisoma* in his new family Oligoterorhynchidae Van Cleave, 1931 later becoming Cavisomidae Meyer, 1932 (= Cavisomatidae Petrochenko, 1956). *Cavisoma magnum* was subsequently reported in specimens of adult milkfish *Chanos chanos* (Forsskål) (Chanidae) in the Philippines [6,17]. Some of the information lacking in the original description [18] was addressed in the redescription [2] of specimens from *C. chanos* caught in the southern Philippines. Much remained to be addressed. Milkfish was also found infected with *C. magnum* in the Arabian Gulf. Our collection of 1,450 worms from one flathead grey mullet, *Mugil cephalus* Linn. (Mugilidae) in the Arabian Gulf off the Iraqi coast provided the materials to fully describe *C. magnum* using SEM images, make new systematic observations and metal analysis of hooks, and report histopathology in the mullet intestinal tissue.

## Materials and methods

### Collection

Fishes were purchased at the local fish market in Al-Faw City area in southern Iraq, northwest Arabian Gulf (29°58′33″N 48°28′20″E). The intestine of one of 8 flathead grey mullets, *Mugil cephalus* examined from the Arabian Gulf off the coast of Basrah, Iraq in January and February, 2017 was infected with 1,450 worms. The fish averaged about 120 cm in total length. One 130 cm long milkfish, *C. chanos*, obtained at the same site on November 14, 2017 was infected with about 350 specimens of *C. magnum*. *Chanos chanos* was previously reported as a host of *C. magnum* in the Philippines [6,17].

The intestinal tract was examined under a dissecting scope and many unidentified crustaceans and large acanthocephalans were collected, recorded, and placed in clean plastic bags, chilled, and sent to the Marine Science Center, Basrah University. Worms were stored in 70% ethanol, gross lesions were recorded, and host tissue samples were fixed in 10% neutral buffered formalin. Selected samples were shipped to our Scottsdale, Arizona facility for processing and further studies. All data collected, together with digitized images, were stored on a USB for future analysis and examination, as reported in Amin *et al.* [1].

### Study of specimens

Worms were punctured with a fine needle and subsequently stained in Mayer’s acid carmine, destained in 4% hydrochloric acid in 70% ethanol, dehydrated in ascending concentrations of ethanol (24 hr each), and cleared in 100% xylene then in 50% Canada balsam and 50% xylene (24 hr each). Whole worms were then mounted in Canada balsam. Measurements are in micrometers, unless otherwise noted; the range is followed by the mean values between parentheses. Width measurements represent maximum width. Trunk length does not include proboscis, neck, or bursa. Line drawings were created by using a Ken-A-Vision micro-projector (Ward’s Biological Supply Co., Rochester, N.Y.) which uses cool quartz iodine 150 W illumination. Color-coded objective (10X, 20X, 43X) lenses are used. Images of stained whole mounted specimens were projected vertically on 300 series Bristol draft paper (Starthmore, Westfield, Massachusetts), then traced and inked with India ink. Projected images were identical to the actual specimens being projected. Voucher specimens were deposited in the University of Nebraska’s State Museum’s Harold W. Manter Laboratory (HWML) collection in Lincoln, Nebraska, USA.

### SEM (Scanning Electron Microscopy)

Samples of parasites that had been fixed and stored in 70% ethanol were processed following standard methods. These included critical point drying (CPD) in sample baskets and mounting on SEM sample mounts (stubs) using conductive double-sided carbon tape. Samples were coated with gold and palladium for 3 minutes using a Polaron #3500 sputter coater (Quorum (Q150 TES) www.quorumtech.com) establishing an approximate thickness of 20 nm. Samples were placed and observed in an FEI Helios Dual Beam Nanolab 600 (FEI, Hillsboro, Oregon). Scanning Electron Microscope with digital images were obtained in the Nano lab software system (FEI, Hillsboro, Oregon). Images were taken at various magnifications. Samples were received under low vacuum conditions using 10 KV, spot size 2, 0.7 Torr using a GSE detector.

### X-ray microanalysis, EDAX (Energy Dispersive Analysis for X-Ray)

Standard methods were used for preparation, similar to the SEM procedure. Specimens were examined and positioned with the above SEM instrument, which was equipped with a Phoenix energy-dispersive x-ray analyzer (FEI, Hillsboro, Oregon). X-ray spot analysis and live scan analysis were performed at 16 kV with a spot size of 5, and results were recorded on charts and stored with digital imaging software attached to a computer. The TEAM *(Texture and Elemental Analytical Microscopy) software system (FEI, Hillsboro, Oregon) was used. The data included weight percent and atom percent of the detected elements following correction factors.

### Ion sectioning of hooks

A dual-beam SEM with a gallium (Ga) ion source (GIS) was used for the LIMS (Liquid Ion Metal Source) part of the process. The hooks of the acanthocephalans were sectioned using a probe current between 0.2 nA and 2.1 nA according to the rate at which the area is cut. The time of cutting is based on the nature and sensitivity of the tissue. Following the initial cut, the sample also goes through a milling process to obtain a smooth surface. The cut was then analyzed for chemical ions with an electron beam (Tungsten) to obtain an X-ray spectrum. The intensity of the GIS was variable due to the nature of the material being cut.

### Histology

**Figures 20-25:**
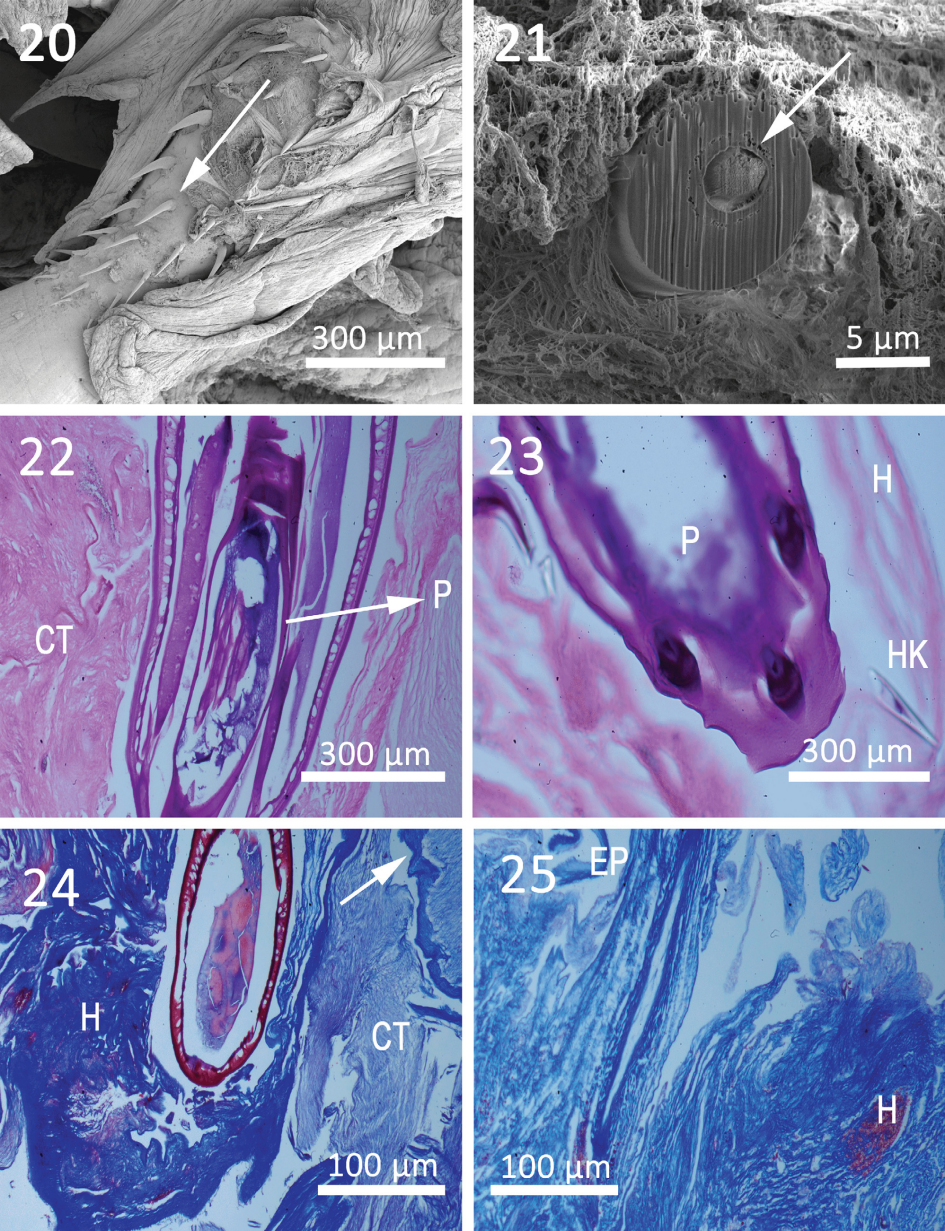
Histopathology of *Cavisoma magnum* in the intestinal track of *Mugil cephalus* from the Arabian Gulf. 20. SEM of attached worm; note hooks (arrow) on the proboscis of a worm. This image shows the gross pathology and the extreme damage to host intestinal tissue. 21. Gallium-cut hook (arrow) from proboscis of attached worm. Note host connective tissue surrounding worm. 22. Proboscis (P) of a worm. Host connective tissue (CT) is visible with remnants of the intestinal epithelium next to the worm. 23. Proboscis (P) of a worm with sections of worm hooks (HK) and host tissue (H) surrounding worm. 24. Trichrome preparation of infected host tissue (H) section and worm body are visible. Note remnants of host intestine (arrow). 25. Area where worm had infected the host tissue (H). Note ports of hemorrhaged blood caused by worm penetration and remnants of the host intestinal epithelium (EP).

Infected host tissue was fixed in 10% buffered formalin and after dehydration and blocking, the specimens were processed using standard methods [3,13]. The paraffin blocked tissue was sectioned at 4–6 microns, placed on glass slides and stained with hematoxylin and eosin (HE). Additional sections were stained with Mallory’s trichrome to emphasize pathological responses to the parasite [9]. The prepared glass slides were viewed with an LSM laser (Carl Zeiss, Thornwood, New York) equipped compound light microscope with representative pictures taken at varying magnifications with a digital camera. HE is a standard stain for tissue, whereas Mallory’s trichrome helps differentiate granular tissue typical of parasite invasion. The histopathological sections (Figs. 20-25) were selected from a much larger collection of sections on 85 glass slides in RAH’s collection.

## Results

The prevalence of infection in the grey mullet in our study was low, 1 of 8 fish. The intensity of infection of one fish with 1,450 large worms was, however, very high. The grey mullets have never previously been reported as hosts of *C. magnum*. The finding of this worm in the Arabian Gulf is also a new and distant geographical record.

Our specimens from grey mullet in the Arabian Gulf provided more information than those described by Southwell [18] and Arthur *et al.* [2]. The description [18] was incomplete and the redescription [2] corrected many of the earlier problems but had its own inadequacies and oversights, especially regarding the proboscis armature and hook roots, egg anatomy and reproductive system structures in males and females. Differences in the egg shape and the organization of cement glands may also have been related to different host or geographical variables.

### *Cavisoma magnum* (Southwell, 1927) Van Cleave, 1931

Family: Cavisomidae Meyer, 1932

Genus: *Cavisoma* Van Cleave, 1931

Type host: Sea bass, *Serranus* sp.

Other hosts: Spotted surgeonfish, *Ctenochaetus strigosus* (Bennett) (Perciformes) [15,18], milkfish *Chanos chanos* (Forsskål) (Chanidae) [2] and this paper, and *Siganus lineatus* Valenciennes (Siganidae) [7], Grey mullet, *Mugil cephalus* Linn. (Mugilidae) (new host).

Site of infection. Intestine.

Type locality: Indian Ocean “off Negapatam, Ceylon” [=Negappattinam, India].

Other localities: From an unspecified locality off Ceylon (Sri Lanka) [15,18], the Red Sea [15], Basilan Strait off Zamboanga, Zamboanga del Norte Province, Mindanao Island, Philippines [2], and New Caledonia, South Pacific [7], Al-Faw City area in southern Iraq, northwest Arabian Gulf (29°58′33″N 48°28′20″E).

Specimens deposited: HWML collection no. 139340. Redescription (Figs. 1-19); specimens from *Mugil cephalus*, northwest Arabian Gulf.

**Figures 1-7:**
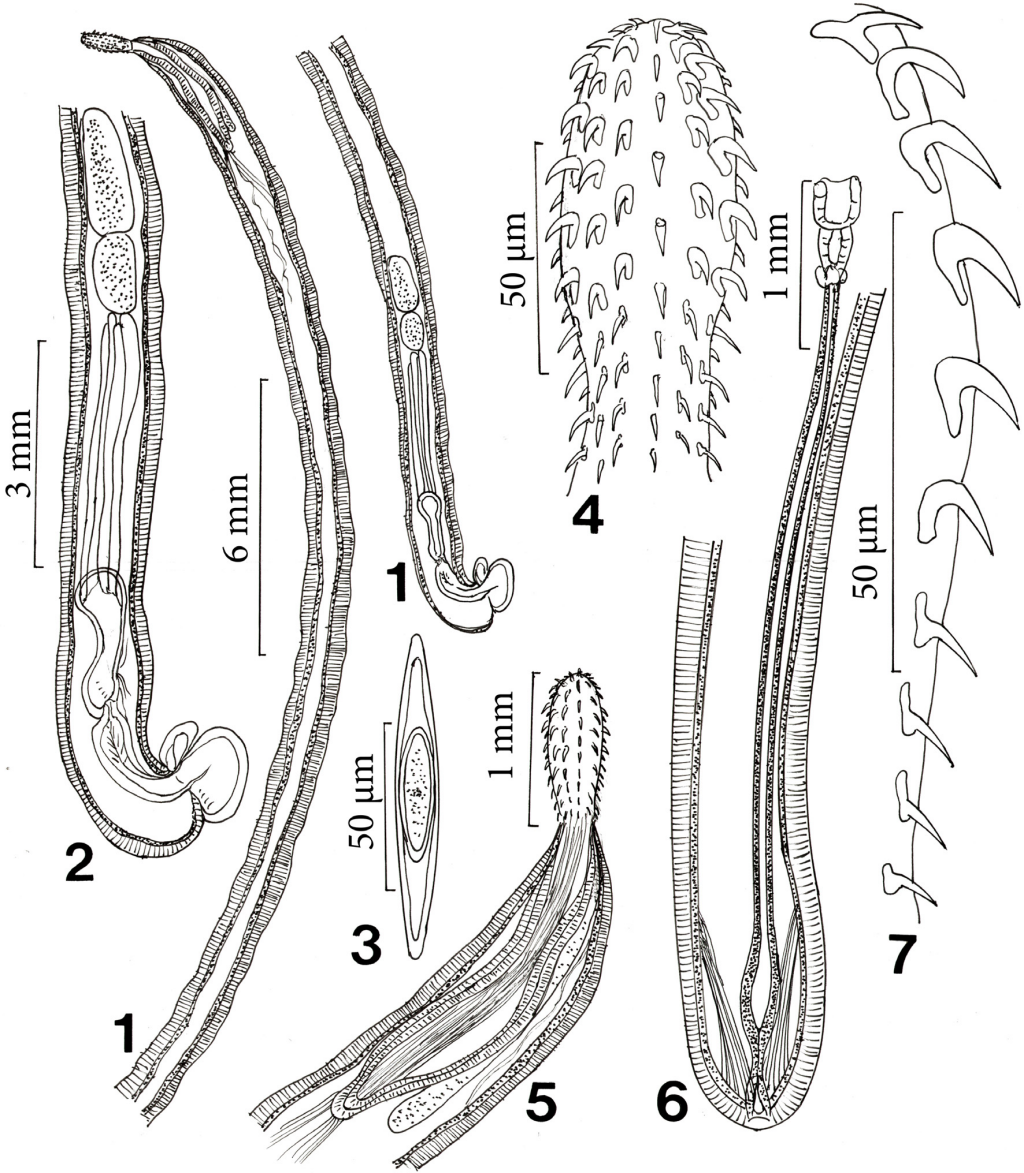
Line drawings of specimens of *Cavisoma magnum* collected from *Mugil cephalus* in the Arabian Gulf. 1. A male specimen. Note the thick body wall here and elsewhere. 2. The posterior part of the same male in Figure 1 showing the reproductive system. 3. A ripe egg removed from the body cavity. 4. The proboscis of an adult male. 5. The anterior part of the trunk of a male specimen showing the relationship in size and shape of the proboscis, receptacle and lemnisci. 6. Posterior part of the trunk of a female specimen showing the reproductive system. Note the simplified vagina, long uterus, and the two para-vaginal bundles of fanning fibers. 7. One row of proboscis hooks showing three types of hooks/roots, the anterior-most hook with lateral hook with prominent manubrium, the regular subapical hooks with posteriorly directed roots, and the posterior hooks with root stubs and anterior manubria. The hooks and inter-hook spacing are identical to those in actual specimens.

**Figures 8-13:**
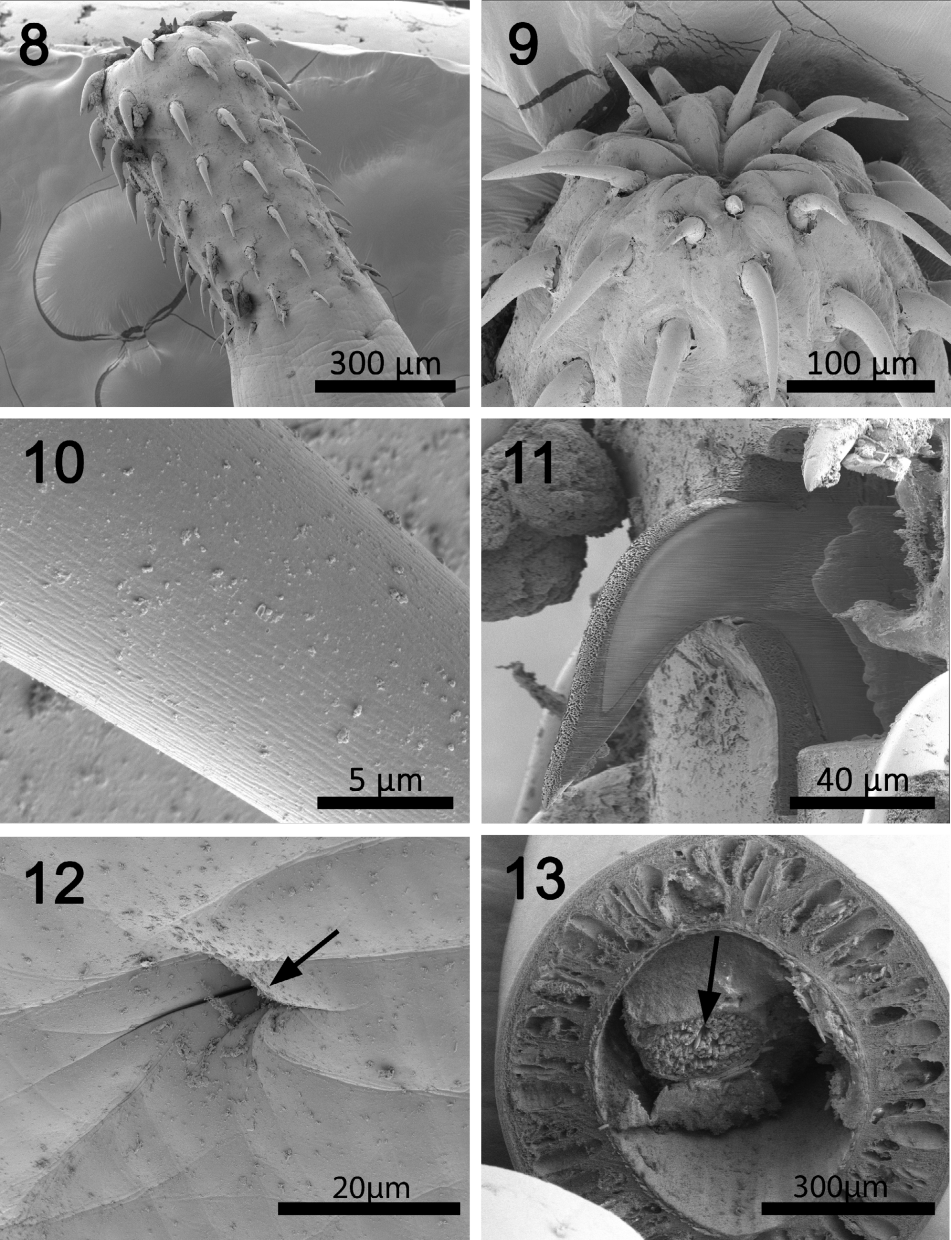
SEM of specimens of *Cavisoma magnum* collected from *Mugil cephalus* in the Arabian Gulf. 8. A lateral view of a proboscis not fully extended showing one lateral neck sensory pore (arrow). 9. A slightly invaginated apical end of a proboscis showing retracted epidermal cone. 10. A high magnification of a proboscis hook showing the shallow longitudinal serrations on the surface of hooks. 11. A longitudinal gallium cut of a proboscis hook showing the thick sulfur-rich hardened areas at the hook tip and edge (Table 3). 12. High magnification of the neck sensory pore shown in Figure 8. 13. A cross section of a gravid female showing the thick tegument and lacunar canals and a mass of eggs within the ligament sac in the body cavity (arrow).

**Figures 14-19:**
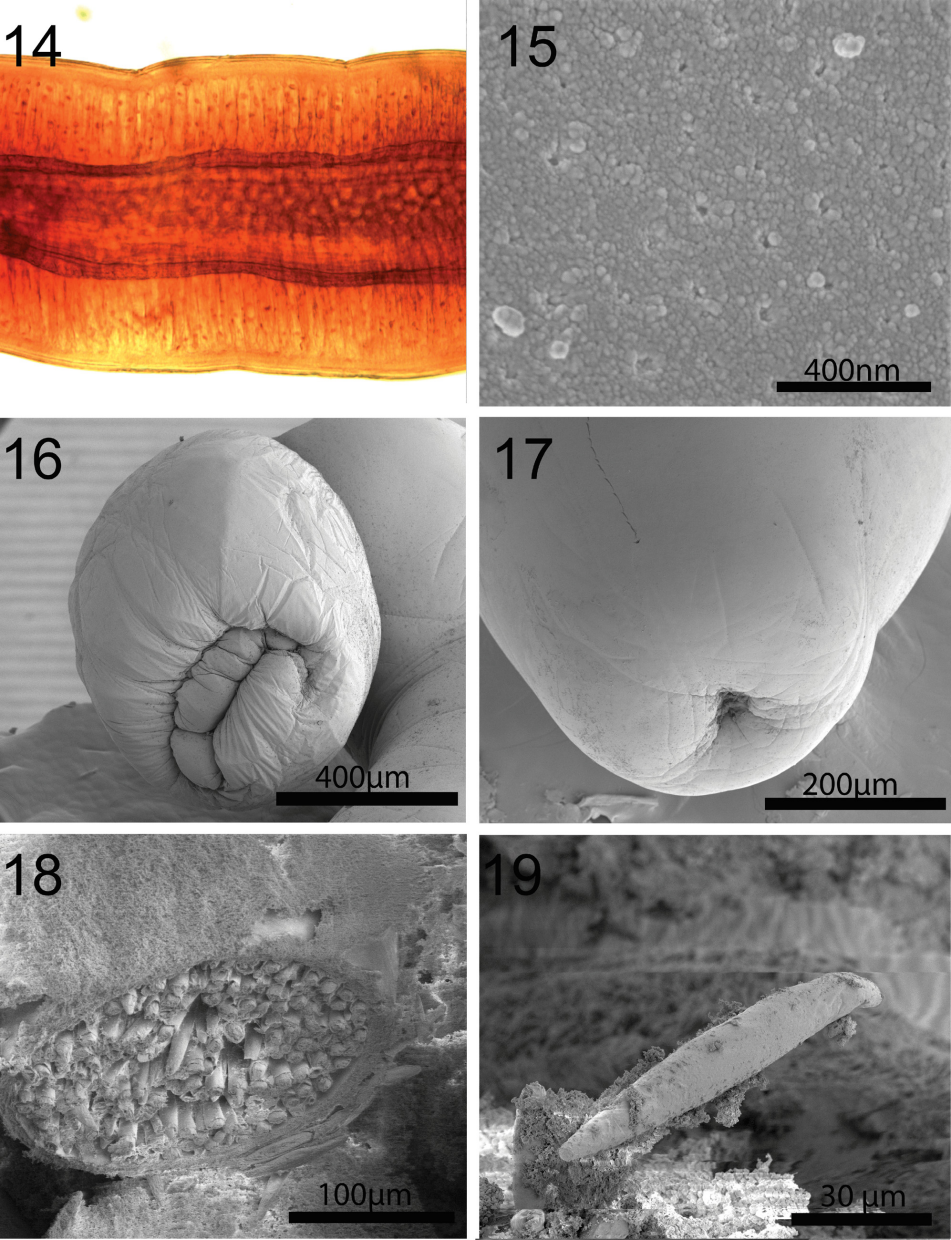
SEM and microscopic images of specimens of *Cavisoma magnum* collected from *Mugil cephalus* in the Arabian Gulf. 14. A microscopic image of a section of a female specimen showing the outer cuticle, thick tegument and the darker internal circular muscle layer. 15. Epidermal micropores in the posterior trunk section of a worm. 16. A basal perspective of a bursa showing its thick unornamented muscular organization with invagination of its inner orifice. 17. A ventro-lateral view of the terminal female gonopore. 18. A high magnification of packed eggs in the ligament sac of a gravid female. 19. A fully developed ripe egg.

#### Redescription of specimens from *Mugil cephalus* (Figs. 1-19)

**Figure 26:**
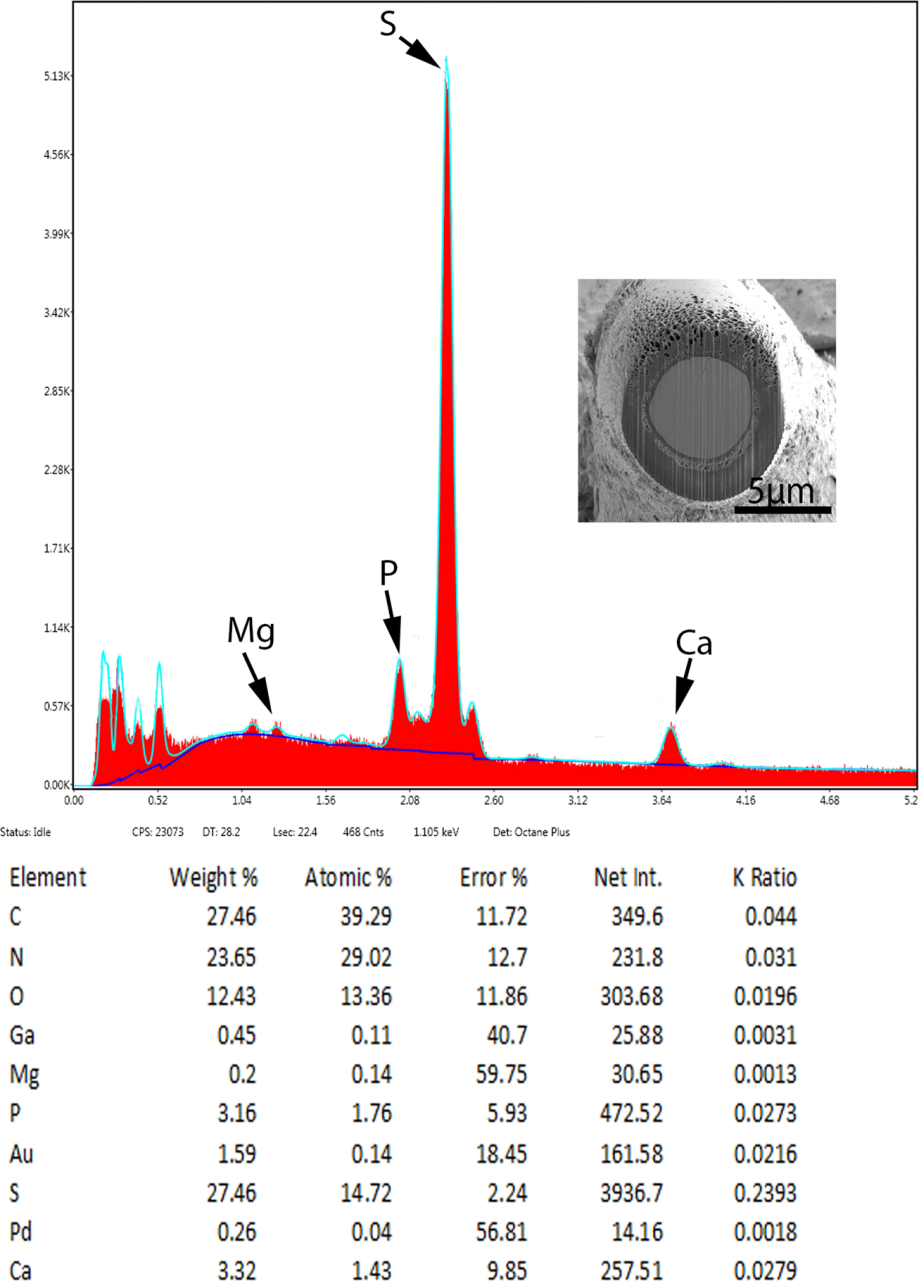
X-ray elemental scan (XEDS) of a *Cavisoma magnum* hook. Edge of a gallium cut showing high sulfur content. Insert: SEM of cross gallium cut hook.

*General*. With characters of the genus *Cavisoma*. Trunk long, cylindrical, elongate, without evident pseudosegmentation (Fig. 1) but with notched shallow epidermal annulations. Trunk and shared structures considerably larger in females than in males. Body wall aspinose, with very thick tegument of prominent lacunar system and nucleated cells and well defined inner circular muscle layer (Figs. 6, 13, 14). Epidermis with many micropores (Fig. 15) associated with internal crypts and vary in diameter and distribution in different trunk and other locations. Proboscis oblong, slightly wider in anterior third (Figs. 4, 5, 8), with apical cone, prominent when partially retracted (Fig. 9). Proboscis with 12–13 longitudinal rows of 9–11 hooks each. Cortical surface of hooks with longitudinal grooves (Fig. 10) and with thick sulfur-rich hardened layers at the hook tip and edge (Figs. 11, 26). Hooks and roots of 3 types: (1) Apical hook with horizontal root and prominent opposite lateral manubrium. (2) Next 5 hooks with strong simple, posteriorly directed roots. Fourth hook in a row from anterior most robust (thickest diameter at base) with longest blade and root. (3) Posterior 4 slender hooks with weak abbreviated roots (stubs) and long faint anterior manubria (Fig. 7). Basal hook smallest. Neck prominent, with 2 sensory pores (Figs. 8, 12). Proboscis receptacle double walled, narrowing at posterior end, with cephalic ganglion near its middle. Lemnisci digitiform, usually but not always somewhat shorter than receptacle (Figs. 1, 5).

*Male* (based on 17 mature adults with sperm). See measurements and counts in Tables 1 and  2. Reproductive system at posterior end of trunk (Fig. 1). Testes oval, in tandem with anterior testis larger than posterior testis. Cement gland 4, anterior gland longest, usually bent anteriorly at posterior end of posterior testis, terminating posteriorly at anterior end of Saefftigen’s pouch. Saefftigen’s pouch massive, bulboid anteriorly and cylindrical posteriorly (Figs. 1, 2). Bursa round without specialized structures, pores or discs (Fig. 16).

**Table 1:** A comparison of morphometric accounts of *Cavisoma magnum.*

	Southwell, 1927 [18]	Arthur *et al.*, 1995 [2]	This paper
Males	N=20 males & females	N=15	N=17
Trunk L x W	up to 36.00 × 1.00	19.42-51.36 (35.37) x 0.73-1.53 (1.25)*	29.25-48.75 (39.20) x 1.02-1.35 (1.18)
Neck L x W	–	0.25-0.40 (0.31) x 0.46-0.50 (0.48)	0.26-0.52 (0.42) x 0.42-0.55 (0.51)
Proboscis L x W (0.36)	1.10 × 0.45	0.91-1.05 (0.97) x 0.33-0.48 (0.39)	1.02-1.27 (1.13) x 0.32-0.45 (0.36)
Hooks			
Long. rows x hooks/row	12 × 8-10	12-13 × 9-10	12-13 (12.6) x 9-11 (10)
Longest & basal/hooks	ca 110 & 70 μm	105-125 (116) & 70-90 (80) μm	125-127 (126) & 67-92 (85)
Receptacle L x W	2.60 x −	1.23-3.09 (2.60) x 0.34-0.52 (0.44)	2.37-3.55 (3.00) x 0.26-0.50 (0.39)
Lemnisci L x W	shorter than proboscis	1.46-4.05 (12.60) x 0.25-0.58 (0.37)	1.66-3.75 (2.75) x 0.09-0.27 (0.17)
Ant. testis L x W	1.17 × 0.10	0.70-2.53 (1.42) x 0.25-2.09 (0.56)	1.12-1.87 (1.35) x 0.30-0.52 (0.40)
Post. Testis L x W	1.03 × 0.10	0.60-1.49 (1.04) x 0.24-0.94 (0.51)	0.75-1.62 (1.02) x 0.30 × 0.55 (0.44)
Cement gland L x W	3.25 x −	1.52-4.49 (2.99) x 0.06-0.27 (0.17)	1.70-4.62 (2.79) x 0.06-0.25 (0.16)
Saefftigens pouch L x W	–	1.28-2.85 (2.16) x 0.39-0.98 (0.62)	1.37-2.37 (2.02) x 0.45-0.57 (0.50) anteriorly
(0.32) posteriorly			
Females	N=20 males & females	N=16	N=18
Trunk L x W	up to 70.00 × 1.50	10.67-48.93 (30.21) x 0.54-1.89 (1.11)	37.00-66.25 (50.95) x 0.95-1.75 (1.36)
Neck L x W	–	0.19-0.44 (0.25) x 0.43-0.57 (0.50)	0.31-0.57 (0.45) x 0.42-0.73(0.58)
Proboscis L x W	1.10 × 0.45	0.79-1.08 (0.95) x 0.32-0.43 (0.37)	1.04-1.25 (1.14) x 0.34-0.43 (0.38)
Hooks			
Long. rows x hooks/row	12 × 8-10	12 × 8-9	12-13 (12.3) x 10
Longest & basal hook	110 & 70 μm	105-130 (115) & 65-95 (80) μm	130-146 (134) & 77-104 (91)
Receptacle L x W	2.60 x −	1.57-3.21 (2.30) x 0.23-0.63 (0.42)	2.42-3.55 (3.12) x 0.34-0.50 (0.44)
Lemnisci L x W	shorter than proboscis	1.58-4.14 (2.72) x 0.13-0.44 (0.25)	2.62-3.87 (3.28) x 0.16-0.37 (0.25)
Reproductive syst. L	–	–	5.82-5.87 (5.85)
Eggs L x W	up to 120-130 × 22 μm	103-121 (113) x 15-20 (17) μm	100-120 (106) x 11-21 (15)
Hosts	*Ctenochaetus strigosus serranus* sp	*Chanos chanos*	*Mugil cephalus*
Locality	Indian Ocean off southern India and Sri Lanka	Basilian Strait, Philippines	Arabian Gulf off Iraq

* Range (mean) length x width in mm, unless otherwise noted.

**Table 2:** Measurements of proboscis hooks and roots of *Cavisoma magnum* from *Mugil cephalus* in the Arabian Gulf.

	Hook length		Hook thickness at base		Root length	
			
Hook no.	Males	Females	Males	Females	Males	Females
1	95-107 (100)*	95-115 (107)	17-30 (24)	20-40 (27)	47-55 (51)**	49-60 (53)
2	110-120 (116)	115-127 (121)	27-42 (34)	27-46 (37)	65-100 (77)	70-110 (88)
3	115-127 (116)	120-132 (126)	30-55 (46)	39-50 (43)	80-112 (93)	80-110 (96)
4	125-127 (126)	130-146 (134)	40-47 (43)	45-52 (51)	87-120 (100)	95-120 (105)
5	124-125 (124)	117-135 (129)	30-40 (35)	40-52 (45)	80-87 (83)	95-114 (100)
6	112-120 (117)	112-138 (122)	20-30 (25)	32-42 (38)	62-75 (70)	75-97 (89)
7	97-117 (109)	107-130 (119)	17-20 (19)	20-27 (25)	–***	–
8	87-112 (105)	92-125 (109)	15-20 (17)	22-30 (23)	–	–
9	75-107 (93)	87-114 (99)	15-17 (15)	20-22 (21)	–	–
10	67-92 (85)	77-104 (91)	12-15 (13)	15-22 (18)	–	–

* Range (mean) in μm in 4 males and 4 females.

** Anterior hook root with anterior manubrium about as long as root oriented laterally.

*** Roots of posterior 4 hooks are abbreviated but have long anterior manubria.

*Female* (based on 18 specimens, most with eggs). See measurements and counts in Tables 1 and  2. Genital pore terminal (Figs. 6, 17). Two prominent sets of ligaments originating near vagina and fanning anteriorly (Fig. 6). Reproductive system 11.4% length of trunk. Eggs fusiform with long polar ends and polar prolongation of fertilization membranes (Figs. 3, 19), usually enclosed within ligament sac (Figs. 13, 18).

#### Remarks

The incompleteness of the information provided in Southwell [18] is reflected in the missing information in Table 1. Additionally, longest hook length in his specimens (sex not stated) was 110 compared to 146 in some of our female specimens. The size of other structures in his material, e.g., proboscis, receptacle, and anterior testis, was also markedly smaller than in our specimens. His specimens were “pseudo-annulated… (Fig. 1) …..probably as a result of contraction.” The proboscis in his material was club-shaped bearing 8–10 hooks per row. The proboscis in our specimens was more oblong and with more hooks per row (9–11). The only other descriptive account of *C. magnum* is that of Arthur *et al.* [2], whose redescription from *Chanos chanos* in the Philippines is a considerable improvement over the original description, but varies from ours in the following points. Arthur *et al.* [2] report 8–10 proboscis hooks per row but their figure 2 shows some hook rows with 6 hooks each. The angle of the single hook root (their fig. 3) is distant from the blade. The egg (their figure 4) appears oblong; the outer shell is actually considerably more prolonged at poles. The female reproductive system (their figure 5) lacks the 2 sets of fanning ligaments originating near the vagina. The posterior end of their male specimen (their figure 6), showed severe pseudo-segmentation in disagreement with their text description. Most significantly, they describe the “Four basal-most hooks in each row” as “rootless.” These hooks are actually rooted and the roots have prominent anterior manubria but they are faint and hard to find. Their description makes no reference to the 3 kinds of hook roots of the apical hook, of the other rooted hooks, and of the 4 posterior spiniform hooks. The cement glands do not often reach “the midlevel of Saefftigen’s pouch” as stated, and are bundled rather than separate (their fig. 6).

### Histopathology

The results of the histopathological study in *M. cephalus* are represented by Figures 20 to 25. The initial tissue fixation did not allow immediate worm response analysis for the host. The proboscis becomes embedded into the connective tissue layers of the host intestine (sub mucosa) with host collagenous fibers attached to the hooks (Fig. 20). The gallium-cut hook demonstrated that the collagenous fibers are closely attached to the solid, multi-layered hook (Fig. 21). A tissue section of the hook-lined proboscis is shown in Figure 23, which is everted from the anterior end of the acanthocephalan (Fig. 22). The next two figures show the depth of worm invasion into the sub-mucosal, connective tissue part of the host intestine (Fig. 24). The trichrome stain preparations (Figs. 24, 25) display the amount of connective tissue in the area, extensive host cell necrosis (Fig. 24), hemorrhaging (Fig. 25) and remnants of the epithelial tissue of the host intestinal mucosa (Fig. 25). The host has generated large amounts of connective tissue. The hemorrhaging is primarily due to the destruction of capillary vessels in the host intestine. The worm, due to its large size and invasive properties, appears to be very destructive to the host intestinal tissue.

### X-Ray elemental analysis (EDAX)

**Figure 27:**
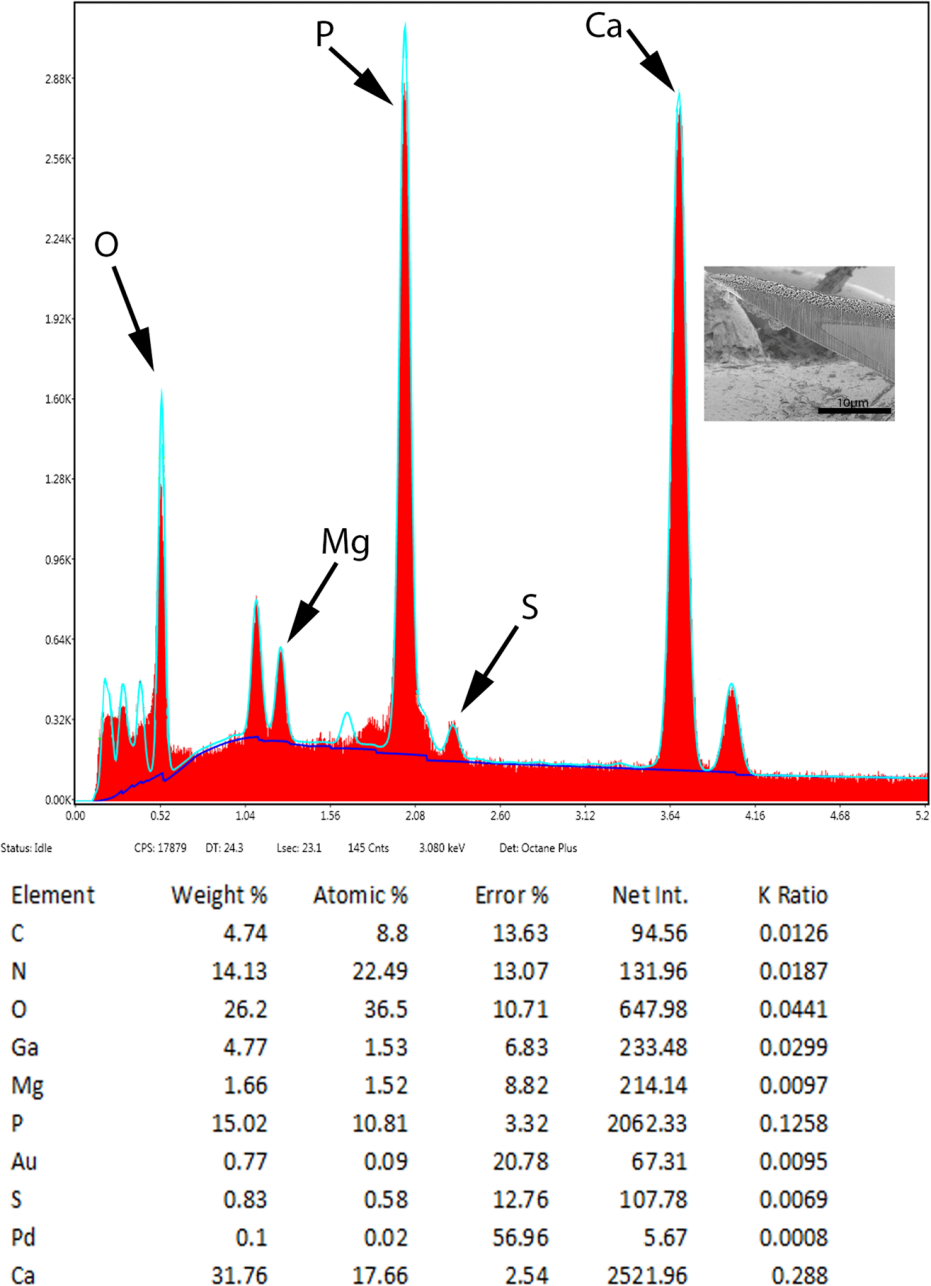
X-ray elemental scan (XEDS) of a *Cavisoma magnum* hook. Center base of a longitudinal gallium cut showing typical levels of phosphorus, calcium and sulfur content. Insert: SEM of a longitudinal gallium-cut hook.

The results of the x-ray elemental analysis are given in Table 3 and Figures 26 and 27. Due to the thickness of the worm body, a scan was taken of that area which demonstrated common protoplasmic elements. Scans were completed for the hook and then 4 positions on the hook cut by a gallium beam. High levels of sulfur were observed in the hook tip (43.51 wt. %) and edges (27.46 wt. %), which is not characteristic of other acanthocephalan hooks. This was also displayed by the overall hook (17.3 wt. %) scan. The center and base of the hook did not have high levels of sulfur but contained mostly phosphorus and calcium, two other essential elements for hook structure (Fig. 27). The thickness of the hook outer layer with high levels of sulfur and solid nature of the hooks is displayed by Figure 11 and by the spectrum print out (Fig. 26).

**Table 3:** X-Ray scans for hooks and trunk of *Cavisoma magnum* from *Mugil cephalus.*

	Trunk	Hook	Hook tip	Hook mid cut edge	Hook mid cut center	Hook base
P (Phosphorus)	1.20	3.14	4.74	3.16	21.44	15.02
S (Sulphur)	1.68	17.30	43.51	27.46	0.97	0.83
C (Calcium)	0.68	3.34	5.66	3.32	39.30	31.76
Mg (Magnesium)	n**	n	n	n	n	1.66

^*^Four chemical elements are listed by weight percent (wt. %) for area. Common elements in living cells (H, O, N) and coating and cutting elements (Pd, Au, Ga) are not listed.

** n = negligible amount

## Discussion

While the prevalence of infection in the grey mullet from the Arabian Gulf was low (1 of 8 fish infected), the intensity of infection of one fish with 1,450 large worms was very high, suggesting that grey mullets are also natural hosts of *C. magnum,* which has never previously been reported. One milkfish also examined in the Arabian Gulf in November, 2017 was heavily infected with specimens of *C. magnum*. These findings suggest that the Arabian Gulf may be another endemic habitat for this parasite, in addition to the South Pacific and the Indian Ocean where earlier accounts report lighter infections. Southwell (1927) collected 26 worms from two species of bass; 20 worms from *Serranus* sp. and 6 worms from *Ctenochaetus strigosus* (Perciformes) off Sri Lanka; host numbers and month of collection were not reported but the paper was received for publication on March 31, 1927. Arthur *et al.* [2] found 30 and 32 worms in two of 5 examined milkfish off the Philippines on March 23, 1987. One other study of spermiogenesis in *C. magnum* was carried out on 7 specimens collected from one out of 6 (16%) naturally infected golden-lined spinefoot fishes, *Siganus lineatus* Valenciennes (Siganidae), off New Caledonia, South Pacific [7].

The flathead mullet is cosmopolitan in coastal waters of the tropical and temperate zones of all seas at temperatures between 8 and 24 °C [8,14]. It occupies fresh, brackish and marine habitats at depths ranging between 0–120 m over sandy or muddy bottoms and dense vegetation [4], and feeds on zooplankton as juveniles and on algae, detritus and small invertebrates as adults [10]. The continuity of the distribution of *C. magnum* from the Pacific to the Indian Ocean to the Arabian Gulf must include a presence in the Red Sea. A confirmation of this supposition was found in Parukhin [15]. In their 7 extensive expeditions collecting parasites of southern commercial marine fishes from Vietnam, Africa, the Red Sea, the Indian Ocean, and the Mediterranean between 1959 and 1973, an international team of scientists reported *C. magnum* in the Red Sea from the gut of 2 specimens of *Acanthurus* sp. (4–5 worms), as well as from *Serranus* sp. and “*Acanthurus strigosus*” in Sri Lankan waters [15].

### Histopathology

*Cavisoma magnum*, due to its invasive properties, causes extensive damage to the host intestine. The well-equipped proboscis penetrates through the outer host mucosa and attaches to the lower connective tissue submucosa. Collagenous fibers from the host surround the proboscis of the worm and attempt to encapsulate and isolate the acanthocephalan (Fig. 26). Hemorrhaging of capillary vessels and extensive cell necrosis follow the invasive path of the worm. The histopathology results are similar to others described by Amin *et al.* [1] and Heckmann *et al.* [12].

### X-ray scans

The X-ray scans for *Cavisoma magnum* displayed a unique mineralization pattern for the hooks with excessive amounts of sulfur on the outer layer of the attachment structure (Fig. 13, Table 3). The other major elements for acanthocephalan hooks and protoplasm were present [5,11,19]. The hardened outer layer of the hooks may account for the difficulty for infected host tissue slide preparation. The sulfur ions are found in disulfide bonds linking the amino acid cysteine in the hardened protein. These bonds, in conjunction with Ca and P present in the X-ray scans, form the hardened apatite like mammalian tooth enamel [16]. Using gallium cut hooks, the progression of the hook minerals was followed from the tip to the base of the attachment organ.

## Conflict of interest

The authors declare that they have no conflicts of interest in relation to this article.
